# Vaccine hesitancy and other obstacles to COVID-19 control: lessons from smallpox

**DOI:** 10.11604/pamj.2021.40.1.31356

**Published:** 2021-09-01

**Authors:** Robert Davis

**Affiliations:** 1 Red Cross, Nairobi, Kenya

**Keywords:** COVID 19, smallpox, vaccine

## Abstract

The world confronts today a disease which was unknown as recently as early 2019. Now that there is a safe and effective vaccine against COVID-19, lessons can usefully be drawn from previous well documented vaccination efforts. Of these, the best documented and most successful is the Smallpox Eradication Program (SEP). A review was made of publications by major players in smallpox eradication, respecting the important differences between the disease, this review revealed several points of connection. Cultural factors loomed large both in the eradication of smallpox and progress, to date, in the control of COVID-19. Other points of similarity included political commitment, the set-up of strong surveillance mechanisms, and assurance of uniformly high quality vaccines tested and approved by the World Health Organization. The future of COVID-19 control depends, in part, on lessons learned from previous vaccination efforts. A review of those efforts will avoid repetition of past errors and permit adoption of best practices from the past. Such analyses must, of course, respect the important differences between COVID-19 and smallpox.

## Commentary

The years since 1980, when smallpox was declared eradicated, have seen many initiatives against vaccine preventable diseases. However, the smallpox eradication program (SEP) is the best documented of these, and uniquely in the annals of public health, the one program in the history of public health to have eradicated a disease of humans. It presents many contrasts with COVID-19 control, which we disregard at our peril, but a few points of similarity. Since we want COVID-19 vaccination to succeed, let us look at how and why SEP succeeded.

**Cultural factors:** the role of cultural factors in stopping smallpox transmission is key. Smallpox needed understanding in its cultural context. When the safe and effective smallpox vaccination was introduced, there was pushback from the practitioners of traditional variolation, some of whom derived cash income from that dangerous practice. There was even a smallpox goddess, Chitala, to whom observant Hindus prayed for protection against smallpox. The Yoruba people of Nigeria had their own smallpox deity, to whom they prayed for relief from what they believed, correctly, to be an incurable disease. The practice of some cultures in handling the mortal remains of the smallpox victim before burial was identified as a traditional practice to be discouraged. When we look at the prominent role of antivaxxers in today´s opposition to COVID-19 vaccination, we discover attractive but deceptive narratives of “Nature´s Way” to prevention, based on unproven nostrums which have found widespread acceptance of those with a fear of allopathic medicine.

The best way to combat this narrative is a counter-narrative. In Thailand, where I first arrived in 1969, I was pleasantly pleased to see that there was no smallpox. I later learned from DA Henderson that the Thais had set up smallpox vaccinations in district (amphoe) capitals, long before the creation of the primary health care structures with which Thailand is now endowed. Thai culture places a high value on a clear complexion, and so great was the aversion to the scarring associated with smallpox that the parents of the newborn child would, at great expense and inconvenience, take her/him to the district hospital for vaccination. In Thailand, it was the cultural aversion to pockmarks which motivated parents to get their newborns vaccinated against the disfigurement of variola major. The Thai example shows that culture can be an enabler of public health programs.

**Choice of strategy:** what is needed? The choice of the right, culturally and technically appropriate strategy. Here, the work of William Foege and Donald Hopkins is relevant. Bill Foege discovered that he was up against hierarchical structures which impeded the implementation of the WHO strategy, surveillance and containment. In “House on Fire,” he recounts the story of a fearless young Indian medical officer who raised his hand in front of a high ranking official from Bihar State. Let Foege tell the story.

“One of the field workers, a young Indian physician, raised his hand. He looked too young even to be a medical school graduate, and he was very thin, the epitome of a dedicated field worker. He did not appear to have the needed gravitas for the moment, and I worried that a mistake was in the making. But the physician stood and, with great deference, addressed the minister. He was shaking as he described himself as just a poor village man. But, he said, when he was growing up, there were things you could depend on. For example, if a house is on fire in a village, no one wastes time putting water on the other houses, just in case the fire spreads. That is the mass vaccination strategy. Instead, as in the surveillance/containment strategy, they rush to pour water where it will do the most good - on the burning house? the minister hesitated and stared at the group for some time. And then the unimaginable happened. He changed his mind on the spot. He said, in a small voice, “I´ll give you one more month.” (House on Fire, pp. 171-172) [1].

If Bihar had reverted to mass vaccination, India´s smallpox program might well have failed. The battle for the right strategy was, like Waterloo, a “closely run thing”. The general reader may wish to consult both Foege´s highly readable book, “House on Fire,” and “The Management of Smallpox Eradication in India,” an equally fine book by Lawrence Brilliant. Nicholas Ward´s co-authored book, “The Eradication of Smallpox from India,” gives an excellent overview of how the world´s most populous democracy succeeded in stopping the virus. The WHO official history, “Smallpox,” by Frank Fenner and colleagues, is now available on the WHO webpage [2].

**Better understanding of transmission:** to stop transmission of any virus, one needs a better understanding of how it is transmitted. We have Wehrle and colleagues to thank for a case study of nosocomial smallpox transmission in a German hospital. The hospital was treating a case of naturally transmitted smallpox in a world traveller who had returned from Afghanistan, where the virus was still endemic. The appearance of secondary cases in the same hospital showed that nosocomial transmission through the air conditioning vents was possible [3].

Luckily, we have learned, early on in the current pandemic, about the role of asymptomatic carriers in COVID-19 transmission. We have also learned about the role of “super spreader” events in perpetuating community transmission. We need a fuller understanding of nosocomial transmission of COVID-19 and, in particular, mandatory vaccination of hospital staff as the single best measure for prevention of hospital transmission. It is remarkable that vaccination of clinical staffers is not yet universal. We need also to understand the role of the Hajj in disease transmission. The last case of smallpox in mainland Europe was from a Yugoslavian pilgrim returning from Saudi Arabia. Good surveillance by the Yugoslavian authorities limited secondary spread of the smallpox virus. Luckily, Saudi Arabia has learned appropriate lessons. The 2021 Hajj, just concluded, is limited to those residents of Saudi Arabia with documented vaccination against COVID-19 [4].

**Official commitment:** in Uganda, the end of smallpox transmission in 1971 took place just before Idi Amin´s seizure of power. For 8 long years, the field marshal did what he could to damage the basic health services built up under his civilian predecessors. But by 1971, smallpox had been eradicated. Good timing, and good work on the part of the field marshal´s civilian predecessors. In today´s Tanzania, president Magufuli initially denied the presence of COVID-19 in his country. Ironically, he succumbed, probably to the virus, as did the vice-president of Zanzibar [5]. Under his successor, president Suluhu, the Tanzanian state has begun to cooperate with WHO and COVID-19 Vaccines Global Access (COVAX) to set up a program of prevention, including vaccination, which is technically and culturally suited to the needs of the country. Epidemiological surveillance complements community prevention measures.

**Surveillance:** at the international level, the reorientation of SEP passed, though by a narrow margin, in the World Health Assembly, the governing body of the World Health Organization. After a decade of failure, there was widespread scepticism about the feasibility of global eradication. The task of overcoming scepticism fell to the WHO SEP secretariat, to the Centers for Disease Control, and to the governments of the endemic countries. After 1965, SEP saw a shift in focus towards a surveillance-based program, with an emphasis on case finding as the mainstay of interrupting transmission [6]. In the context of COVID-19, initial missteps with lab tests have been overcome. Both developed and developing countries have, with few exceptions, been able to set up mechanisms for rapid reporting to WHO both morbidity and mortality data on COVID-19. Age-specific reporting of these data has enabled governments to target high risk groups where resources for global vaccination are not yet available.

**Safe and effective vaccines:** one of the first things that WHO did in SEP was to review the state of vaccine manufacturing, especially good manufacturing practice, in the world, with approval of smallpox vaccines for international tenders. Luckily, WHO prequalification for vaccines intended for export is, today, well developed. WHO´s prequalification procedures have placed an effective brake on the export, in international trade, of those vaccines which do not pass muster. A small but effective Geneva based team carefully reviews, and independently evaluates, the dossiers of candidate vaccines for COVID-19, giving the green light for their export if and only if they meet WHO´s criteria for safety and efficacy.

**Lessons learned:** among the lessons learned from SEP, cultural factors may top the list. Most countries now recognize that COVID-19 is not “a little flu”. Disease denialism persists in a few places. Wider dissemination of graphic illustrations of the hospitalized cases of COVID-19 will create demand for the vaccine where it does not exist, and put paid to the “little flu” mythology. The American South is beginning to see the decline in denialism. This decline is associated with widespread interviews of hospitalized victims of COVID-19, who are the most trusted spokespersons for vaccination. The vaccine hesitant who distrust government will trust the eloquent testimony of those who, at death's door, regret their decision to reject vaccination. In Africa, where there is a huge unmet demand for vaccines, only the lifting of the export restrictions on vaccines from other countries will break the logjam in vaccine availability. Nairobi taxi drivers have recounted to this writer their inability to get vaccinated, despite their being members of a high risk group. In most of Africa and much of Asia, it is a question of satisfying the huge and unmet demand for vaccines which will cut the rising morbidity and mortality associated with this new virus. Until coverage levels in Asia and Africa match those of the developed world, effective control of COVID-19 will remain a chimera.

Vaccine nationalism is a major obstacle to a rational international approach. The current need is for ramping up vaccine production in developed and developing countries, with all which this implies in terms of political and financial commitments from developed and developing countries. It can be done. The largest actual and potential suppliers of vaccine to the developing world are, respectively, India and China. In those and other countries, ramping up of existing capacity, along with assurance of good manufacturing practice, can unblock the vaccine supply logjam which currently inhibits expansion of vaccination efforts in most developing countries.

**Caveats:** though there are lessons to learn from SEP, no one should take a cut and paste approach in applying SEP lessons to COVID-19 control. The differences between COVID-19 and smallpox are obvious even to the neophyte: different case: infection ratios, role of asymptomatic transmission with COVID-19 only, and contrasting cold chain requirements for the vaccine, to mention but three. That said, the selective adoption and adaptation of appropriate lessons from SEP can, in today's world, provide useful guidance to national and international decision makers.
